# Implementing informed consent in federated medical research: a blueprint designed to safeguard data subject rights in Germany

**DOI:** 10.1186/s12967-026-08437-y

**Published:** 2026-06-23

**Authors:** Christopher Hampf, Astrid Wiebke Pley, Peter Penndorf, Frank Michael Moser, Stefan Lang, Patrick Werner, Anika Kästner, Torsten Leddig, Dana Stahl, Wolfgang Hoffmann, Martin Bialke

**Affiliations:** 1 Institute for Community Medicine, Department Epidemiology of Health Care and Community Health, University Medicine Greifswald, Ellernholzstr. 1-2, 17475 Greifswald, Germany; 2 Trusted Third Party of the University Medicine Greifswald, Ellernholzstr. 1-2, Greifswald, 17475 Germany; 3 Gefyra GmbH, Otto-Hahn-Str. 9, 48161 Münster, Germany

**Keywords:** Federated consent management, Informed consent, Objection, Withdrawal, GDPR, Patient rights

## Abstract

**Background:**

The establishment of the complementary infrastructures Medical Informatics Initiative (MII) and the Network of University Medicine (NUM) has significantly advanced the medical research landscape in Germany. Both infrastructures focus on the cross-institutional integration of health data as the basis for strengthening medical research, with the MII focusing on the standardization and integration of routine university clinical data and the NUM representing a central infrastructure for clinical studies and data sharing. Within the project ‘NUM Routine Data Platform‘ (NUM-RDP), a federated record linkage approach was implemented across 34 affiliated German university hospitals. Accordingly, a federated Trusted Third Party (fTTP) performed pseudonymization and privacy-preserving record linkage across all the participating sites. This work aims to extend the established fTTP approach to managing consent and withdrawal while considering the current legal frameworks and research initiatives for the use of health data.

**Methods:**

A concept for an extended fTTP, termed ‘fTTP Consent’, is proposed to bridge communication gaps between different sites, facilities and components. This allows for central coordination for the implementation of patients’ consent decisions regarding the storage, transfer and scientific use of their health data in a uniform manner.

**Results:**

Two practical use cases for an ‘fTTP Consent’ have been identified and conceptualized. First, the cross-site improvement of workflows and automated processes should be performed to ensure that consent data are correct in formal, legal, semantic and syntactic terms. Second, the cross-site improvement of automated notification processes for new or updated consent data, including respective withdrawal- and objection-processes, should be performed.

**Conclusions:**

In this study, the ‘fTTP Consent’ has been proposed to reduce communicatory and personnel efforts. The correct and up-to-date realization of data subject rights should be ensured using the NUM-RDP as an example. The designed concept could help to overcome challenges in different consent scenarios (opt-in, opt-out). Furthermore, it could streamline communication and data linkage processes between institutions and countries in future research projects.

## Background

In recent years, the German medical research landscape has changed significantly because of the establishment of the Medical Informatics Initiative (MII) and the Network of University Medicine (NUM). These complementary infrastructural approaches aim to reuse and standardize health data for scientific purposes [1]. The MII focuses on the standardization and integration of routine data provided by and to university clinics. Driven by the COVID-19 pandemic, the NUM was established as a central infrastructure for clinical studies and their data integration across 34 German university hospitals. Both systems provide a foundation for future challenges: the integration of additional external data sources. This is especially important, as data provided by university clinics might not suffice to provide a holistic foundation for scientific research. Much important information and care data, such as cross-sectional care across healthcare providers and services, treatment costs, long-term outcomes, and comorbidities, cannot be fully captured by individual clinical systems. Therefore, integrating external data sources, such as public and private health insurance providers, cancer registries, and various medical registries, can help build a comprehensive foundation for medical research [2]. Data integration across institutions and data sources is therefore vital for future research and patient care.

Under the legal requirements of the General Data Protection Regulation (EU GDPR), the processing of personal medical data is generally prohibited unless a specific legal basis exists (opt-in), such as the patient’s informed consent (IC) [3]. For this purpose, a person must be given the opportunity to ask questions to fully understand the data processing steps before consenting to the specific scientific use of their health data. In an opt-in scenario a provided consent may be withdrawn by patients and study participants at any time without providing reason (GDPR Art. 7 (3)).

But the scientific use of health data is not limited to opt-in approaches based on a patient consent. Legal frameworks on a European and national level exist, allowing the use of health data without explicit prior consent of the data subject (opt-out). An example for an opt-out-based scenario is the European Health Data Space (EHDS) Regulation [4], which is a central prerequisite for future use and exchange of health care data in Europe. At a national level, examples of opt-out regulations include the German Act to Accelerate the Digitalization of the Healthcare System (Digital Act – DigiG) [5] and the German Health Data Utilization Act (GDNG) [6]. The DigiG in particular fosters the implementation of an electronic patient record (ePA) in Germany [7]. In these cases, data subjects generally have the right to object to data processing and usage without stating specific reasons.

Currently, coordinating patients’ consent status across sectors and institutions in a timely manner is a challenge. It demands timely, personnel and communicatory efforts. Ensuring that the patient´s data rights are protected is crucial.

In this paper, the preparatory work provided by the MII and NUM Routine Data Platform (RDP) is described. The current challenges of the NUM-RDP in the context of data transfer and corresponding coordinative and communicatory efforts during the implementation of the patients’ will are explored. Furthermore, general requirements and information flows to improve the status quo are discussed.

### Previous work of the medical informatics initiative germany (MII)

The MII’s standardized broad consent approach was developed [3] by the MII Working Group (WG) Consent and coordinated with the responsible data protection authorities of the federal states. This broad consent is not limited “*to certain diseases or areas of research*” [3]. The modular MII template text was technically implemented by the MII Task Force Consent Implementation (TFCI). The ensuing and already published concept design defines the semantics of consent data [8]. Object identifiers (OIDs) were implemented in the open-source platform ART-DECOR to unambiguously identify a patient’s decision or stated will (specified as ‘consent policies’ [9]). For example, a consent policy might aim to allow the collection or sharing of health data. The MII WG Consent notes [10] that this developed solution may be used only for the MII Broad Consent. It is not adoptable for other research projects in this exact manner.

Within the MII, processes are primarily decentralized, with consents and withdrawals being handled on a site-specific basis. As a result, individuals who participate at multiple sites are required to provide separate consent at each site, and in the case of withdrawal, they must withdraw their consent separately for each site. Consequently, the consent status may differ across participating sites.

### Previous work of the NUM-RDP

NUM-RDP established the necessary infrastructures and processes for the central collection of research data from 34 NUM sites. The MII-Broad Consent [3] accompanied by an additional NUM-RDP-specific consent module provides the legal basis for data processing and research. Within the NUM-RDP, consents are also valid only locally. The situation differs in the case of withdrawals. A project-specific decision (September 2021) states that withdrawals are valid across sites. A withdrawal given by one patient is as valid at one study site as at another. However, a cross-site, uniform, centralized unit supporting necessary withdrawal workflows was not intended to be established within NUM-RDP.

The NUM-RDP infrastructure supports centralized data integration. Each of the 34 NUM sites operates a local Trusted Third Party (TTP) and a local Data Integration Centre (DIC). This local TTP is responsible for the management of person-identifying information, the generation of pseudonyms (PSN) and the management and validation of patient consent at a specific site. The local DIC manages the patient’s health data. A federated Trusted Third Party (fTTP) is centrally used for cross-site record linkage and cross-site pseudonymization within NUM-RDP [11]. In this process the ‘fTTP probability’ is a key component. It performs a privacy-preserving record linkage (PPRL) based on bloom filters used to encrypt personally identifiable information. This approach enables record linkage without disclosing the identity of the data subject to the responsible organization [11]. The fTTP does not support consent management processes. Medical data (MDAT) from patients who have given their consent are transferred from local sites to the Routine Data Platform of NUM-RDP called the ‘Central Research Repository’ (CRR) or ‘Data Management Unit’ (DMU). The pseudonymized medical data are transferred through the NUM Transfer Hub (NTH) to the CRR. With the help of the fTTP [11], site-specific pseudonyms are linked (record linkage), and cross-site pseudonyms are generated. The NTH transfers the MDAT with cross-site pseudonyms to the CRR for central data management. Following the recommendations of the data protection concept of the MII for pseudonymization [12], these pseudonyms are specific per person and data usage project. A single person has a certain number of cross-site pseudonyms depending on the number of research projects using health data of this person. Pseudonyms of a person are securely managed within the fTTP until withdrawal of the respective person or the end of the research project.

### Current challenges within/for the NUM-RDP

The following section illustrates current challenges in data transfer and the implementation of withdrawal processes in NUM-RDP. Table 1 summarizes the challenges for the infrastructure components involved (e.g., DIC, NTH, and CRR). In addition, potential consequences for individuals (patients) affected are listed.

#### Data transfer without validation of consent data

Patient consent documents remain at the local site. Their typical quality indicators are completeness, correctness, and legal certainty (cf [13]). For further data processing, those responsible (local TTP) should perform a quality test (verification) to ensure legal compliance with data use for scientific research (cf [8]). Quality assessment generally occurs during the transfer from a paper-based consent form to ‘consent data’, if an informed consent has not been obtained solely with a digital form [14]. The term ‘consent data’ relates to a structured, queryable, machine-interpretable, semantically annotated format representing the patient’s will. Consent data is often documented based on consent policies.

During the decentralized consent collection process the informed (IC) consents are reviewed solely at the local sites. As personnel effort remains with the site, delays between the acquisition of ICs and the extracted consent data may occur [14]. The total expenditure depends on the number of consents obtained and quality tested each year (linear scaling). The structural consent data are stored decentralized within the local TTP and can usually be made available for inquiry by implementing a consent format based on Health Level 7 Fast Health Care Interoperability Resources (HL7 FHIR). If a person has multiple consents at different sites, which is in fact a valid and expected situation within the MII and the NUM-RDP, the consent data are not cross-site compared during the data transfer process to the CRR. This approach might pose a risk of propositional contradiction, as it delegates the responsibility of lawful data processing to the local NUM site. As of 2024, within the NUM-RDP infrastructure, neither the NTH nor the CRR possess sufficient information (all relevant consents and withdrawals of the persons concerned) to ensure the legal permissibility of data sharing.

#### Potentially high coordination effort during the implementation of withdrawal processes

Patient withdrawals must be communicated and processed quickly within the NUM-RDP because of their cross-site nature (cf [15]., p. 51 ff. “Legal Consequences of Withdrawal”). According to the GDPR (Art. 7 (2–3)), there are no formal requirements to withdraw consent; it should be as easy to withdraw consent as to give it. Currently, processing withdrawals within the NUM-RDP project is based on a minimal withdrawal regulation from 2021. The local site’s responsibility is to receive withdrawals and inform the NUM-RDP components about incoming withdrawals. Their processing is divided into six manual steps. These processing steps entail the following: the partner responsible for the fTTP is informed about the patient’s withdrawal via phone (step 1). They then forward it with its corresponding pseudonym to all relevant components (CRR, sites), thus ensuring that no additional data are sent to the ‘CRR’ (step 2). Afterward, the CRR erases all the data corresponding to the cross-site pseudonyms (step 3). This encompasses all the data of one person, as the CRR must not contain site-specific data. The fTTP is informed about the completed data erasure (step 4). In turn, they inform all relevant sites about this process and request the local erasure of the patient’s data (step 5). Finally, the fTTP erases the Master Person Index (MPI) and additional information used for privacy-preserving record linkage to complete the withdrawal process (step 6). These steps are based on analogous communication, mostly via telephone and/or email and manual processes. Any nascent procedure, therefore, requires high communicational and personnel efforts.

**Table 1:** Summary of current challenges and possible risks for technical components and patients

	Challenges and possible risks…
…for technical components (data processor)	• The NTH, CRR and DMU are not able to approve the existence of a valid IC stating the intended processing purpose (labeling cf. Art. 5 (1) lit. b GDPR) and time (lawfully, fairly and in a transparent manner in relation to the data subject). The NTH, CRR and DMU are processing data from the data-sending location under the assumption that this is being done lawfully. • Only with great (communicational) effort component operators can prove the legality of data processing to the supervisory authority (Art 7. (1) GDPR). • Risk of different consent versions existing for one person at different sites, leading to potential content contradiction. • Even if a withdrawal was received by the study site, it is possible to transmit data via the DMU, due to the high communicatory efforts and its lengthy processing time. This might result in unlawful data usage. • An increase in withdrawals will significantly increase the expenditure in cost, time, and personnel. This might increase the risk for scaling problems.
…for patients (affected individual)	• Due to its lengthy processing time, the patient´s right to withdraw (GDPR Art. 7 (3)) may consequently not be processed in a timely manner. Similarly, the reasonable steps a controller has to take, in accordance with the “Right to erasure” (GDPR Art. 17), could be delayed or not processed correctly.

### Improvement of cross-site processes and automatisms is needed

The identified challenges and potential consequences (see Table 1) illustrate the need for more automation and more standardized orchestration of consent-relevant workflows and processes. In terms of risk minimization measures, cross-site processes and automatisms need to be improved. The necessary functionalities comprise two additional use cases:

- *Use Case 1* (UC1): Consistently ensure the correctness and matching of consent data (formal, legal, semantical, syntactical).
- *Use Case 2 *(UC2): Enable the notification of new or updated consent data, as well as implementation of withdrawal processes.

In this manner, across all the participating research sites, the processes for ensuring the legality and transparency of data processing could be improved, and the correctness of the implementation of patient consent could be ensured. Thus, the recency of consent data could be guaranteed, which reduces the risk of unlawful data transfers or data processing.

## Objectives

This article aims to expand existing fTTP approaches. A technical architecture, ‘fTTP Consent’, to support the identified use cases UC1 and UC2 and improve cross-site processes and automatisms in the context of consent management is proposed. The objectives of this article are as follows:

1. Analyzing the requirements for the implementation of a new ‘fTTP consent’ approach that provides technical support for federated consent, withdrawal and objection processes in opt-in and opt-out research scenarios.
2. Developing a solution concept that addresses corresponding cross-site processes and automatisms while taking into consideration the possible future integration of external data sources.

## Methods

An early NUM project highlighted the need for a unified solution concept for handling consent data in federated and cross-project use cases. With a focus on an application within NUM, initial use cases and technical requirements were collected. Potential data flows between NUM-related infrastructural components were discussed with participating stakeholders in the context of a decentralized consent management. Areas requiring clarification of a syntactic, interoperability and semantic nature were identified.

A first conceptual design for a new component ‘fTTP Consent’ to extend existing federated approaches was outlined [16]. Consequently, two fundamental prerequisites for potential implementation were identified. First, a generalized method had to be identified (or developed) to annotate existing and semantically heterogeneous consent data in a context-independent form in order to be able to evaluate this consent data using uniform search and analysis mechanisms despite the heterogeneous background. For this purpose, a working group was constituted, which then proceeded to develop the Semantic Consent Code [17]. Second, the HL7-D Working Group Consent Management was reactivated to broaden the scope of the current Consent Management standard (v1.0) [18]. The revised version of the standard should facilitate the technical representation of withdrawals and objections, enable search queries regarding the consent status of a patient, and accommodate opt-out scenarios with regard to the anticipated EHDS [4] and GDNG [6].

Since MII and NUM evolve continuously, the first conceptual design for an ‘fTTP Consent’ was revised. In particular, potential interaction with the German Portal for Medical Research Data [19] and the DMUs now relevant alongside the CRR was considered. NUM-RDP-related requirements were generalized to enable use of the ‘fTTP consent’ within a MII-related setting.

These findings formed the basis for the subsequent development of a detailed technical concept in 2024, encompassing coordinated workflows and technical constraints. At the same time, the necessary data elements, the required minimum scope of functionality, and interfaces to the relevant components were defined. At the time, the technical concept considered the ongoing specification work for the new version of the HL7 Consent Management standard [20].

**Fig. 1:**
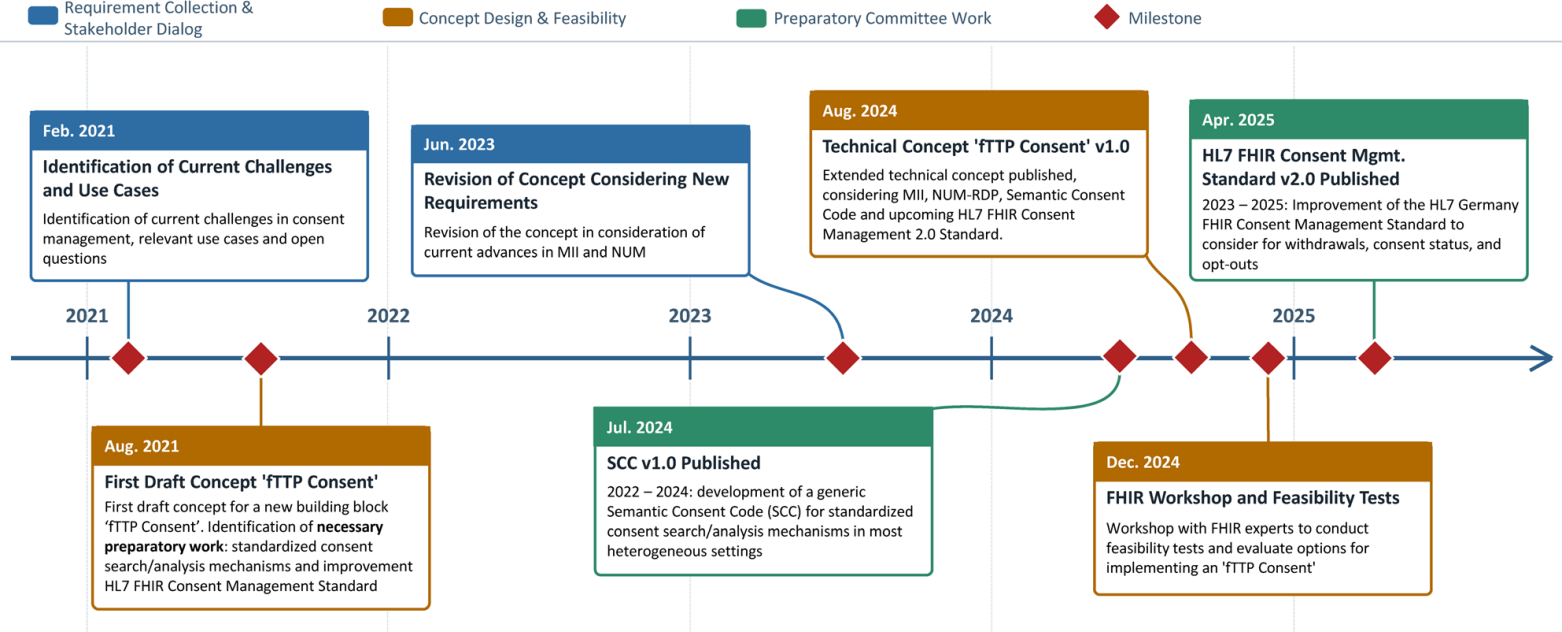
A brief overview of the applied methods to design an ‘fTTP Consent’

As a last step, a feasibility workshop was conducted with a selected FHIR expert, with the aim of evaluating different approaches to implementing ‘fTTP Consent’ prototypically by using HAPI FHIR [21] and customized FHIR operations. The full technical implementation of the ‘fTTP Consent’ is currently pending (as of May 2026). The chronological sequence of the methods applied is summarized in Fig. 1.

## Results

### General requirements

Even though the NUM-RDP adopts an opt-in approach, the solution concept should not be limited only to it. To continue to meet most relevant legal requirements for the use of scientific data in the future, both consent-based (opt-in) and objection-based (opt-out) research scenarios should be supported from the outset. This will promote ongoing research initiatives (MII, NUM) and increasingly relevant data usage scenarios (based on legal frameworks such as EHDS [4], ePA [7] or GDNG [6]).

HL7 FHIR should be the technical basis for communication to maximize the possibility of integrating the new consent component into existing infrastructures. This procedure would be similar to those of the MII and NUM [8]. Furthermore, recent developments and profiling work provided by the HL7 Germany Working Group ‘Consent Management’ must be taken into consideration. This includes the technical representation of opt-in and opt-out scenarios and the urgently required standardization of the definition of consent status and consent documents [20]. For future scenarios, support for project-independent semantic comparability of the processed consent data should be ensured, e.g., based on a ‘Semantic Consent Code’ (SCC) [17] and established standards such as Open Digital Rights Language (ODRL) [22] and common ontologies like Data Privacy Vocabulary (DPV) [23].

### Required information flows

The current infrastructure components in the NUM-RDP were already introduced in detail in the background section. Data is collected and managed in a decentralized approach (DIC: medical data; local TTP: PII; site-specific pseudonym; structured consent). The transmission of health data to a central research platform (CRR: pseudonymized health data) is supported by centralized components (NTH: transmission of health data from site to CRR; fTTP: record linkage and cross-site pseudonymization). The CRR and the DMU are responsible for sharing of health data with research projects.

**Fig. 2:**
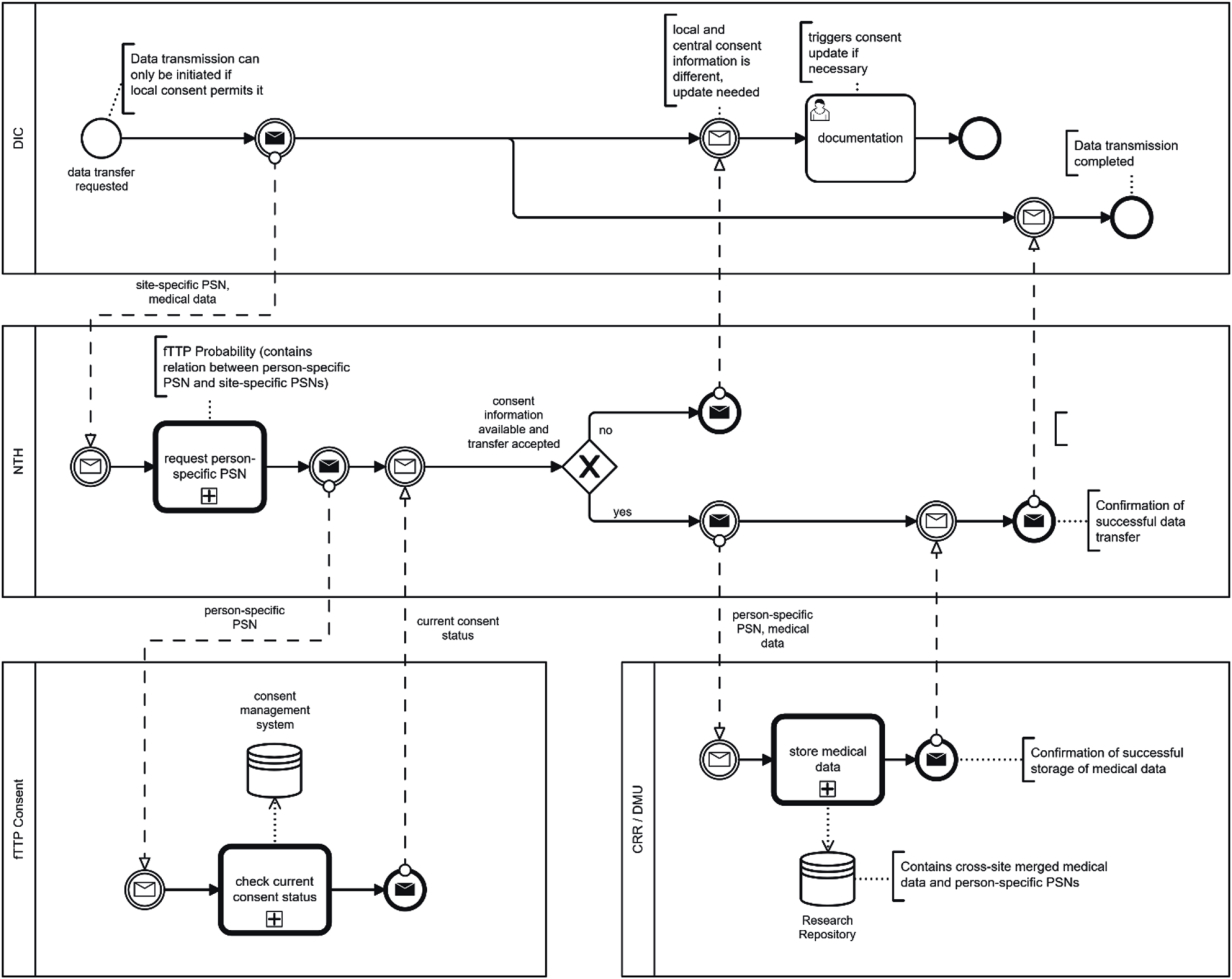
Real-time consent checks prevent legally unauthorized data transfers and thus strengthen the rights of data subjects (UC1). The term “consent” broadly describes the legal admissibility of the intended data processing and applies equally to opt-in and opt-out in this case

**Fig. 3:**
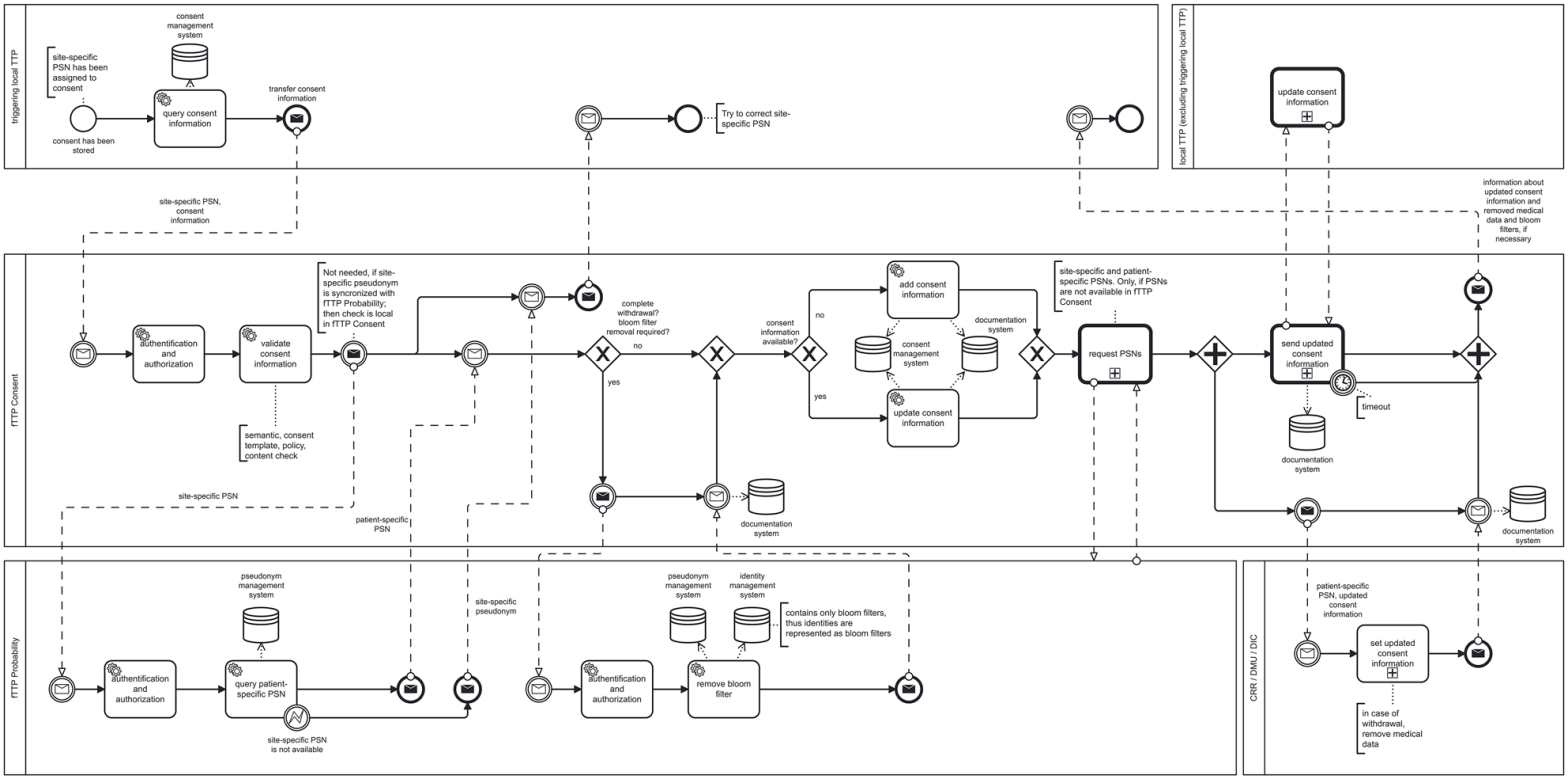
Necessary workflows for the notification of new, updated or withdrawn consent data, including the implementation of withdrawal and objection processes (UC2). Withdrawals and objections may result in the deletion of the personal reference (bloom filter) in the responsible fTTP component (fTTP Probability) [11]

Regarding these current infrastructures and processes in the NUM-RDP and the envisaged use cases UC1 and UC2 for optimizing the status quo, the following figures (Figs. 2 and 3) in Business Process Model and Notation -format (BPMN) describe the nominal state for fulfilling the established objectives.

#### UC1 - data transfer with real-time consent verification

Site-specific pseudonyms are exchanged with cross-site pseudonyms by the fTTP. During this process, the component responsible for the data transfer verifies (NTH) with the ‘fTTP Consent’ whether a valid and context-specific consent has been given by the patient for the data transfer. If the required consent was given, the data are transferred to the CRR/ DMU. Otherwise, data transfer is stopped immediately. The DIC is notified that there is no legal permission to share data. In this way, the risk of a potential violation of legal regulations (by the responsible data processor) is reduced.

#### UC2 - Notification on new, updated and withdrawn/objected consent data, including support for the implementation of withdrawal and objection processes

The ‘fTTP Consent’ is informed about new and updated consent data by the site, which keeps the original documents, scans, and signatures. For communication between the site and the ‘fTTP Consent’, site-specific pseudonyms are used. They are translatable into cross-site pseudonyms within the ‘fTTP Probability’. The ‘fTTP Consent’ documents the most recent consent data in a project-, site-, and person-specific manner. If any information is obliged to be shared with the CRR, e.g., if data transfer is no longer permissible, the ‘fTTP Consent’ will inform the sites accordingly.

If the ‘fTTP Consent’ receives a withdrawal or an objection from a specific NUM site, it documents the request and starts the withdrawal/objection process. All other relevant NUM sites and infrastructure components will be informed and requested to perform their respective tasks, such as inactivating a person’s data at the NUM site. The ‘fTTP Consent’ coordinates and centrally documents those activities and the process statuses. After the sites and NUM components complete their withdrawal/objection tasks, the ‘fTTP Consent’ initiates internal deletion of personal references within the ‘fTTP Probability’ and confirms successful implementation of the withdrawal/objection to the initiating NUM site.

### Data processed within the fTTP consent

For the implementation of an ‘fTTP Consent’ in the sense of a Consent Registry, which includes a central storage of the most recent consent data supported by common management functionalities (CRUD: create, read, update, delete) and search functionalities [24], the information listed in Table 2 and Table 3 can be processed and stored (temporarily) within the ‘fTTP Consent’. In most cases, cross-site pseudonyms are documented to enable communication between the CRR and other components connected to medical data sources. These pseudonyms are essential to link consents to specific individuals and are stored within the ‘fTTP Consent’.

**Table 2:** Necessary data for the processing of a consent registry

Required Data Items	Purpose	Persistency
Site-specific pseudonym	Site-specific pseudonym for the respective person	Permanent
Cross-site pseudonym	Pseudonyms used to attribute a patient’s personal information to their consent data, allowing for complex queries (regarding the consent and withdrawal status) e.g. used to communicate with the CRR	Permanent
Research pseudonym (alias)	Pseudonyms used for data transfers	Permanent
Project-context	Ensures the project context during complex queries	Permanent
Consent-policies/consent-resources	Basis for standardized documentation of consent data across sites. This includes the validity period, a policy’s semantic meaning, and information about consented facts (permit/deny) Scans, signatures, or filled consent documents are explicitly not processed.	Permanent
Validation outcome	For the documentation of a performed consent data validation, and to respond validation results to the respective sites	Permanent

**Table 3:** Necessary data items for the processing of comprehensive withdrawal (opt-in) and objection (opt-out) documentation

Required Data Items	Purpose	Persistency
Withdrawal	Information about received withdrawals (i.e., Person-context, time stamp, site, extent of withdrawal)	Permanent
Process	Progress information about the process and documentation of withdrawal implementation	Permanent
Site-context	Site-context in terms of a site-specific pseudonym	Temporary
Withdrawal confirmation	If a direct notification is necessary, the process confirmations sent by the CRR, local TTP, and DMU will be saved (accountability, traceability, transparency)	Permanent

### The semantics and coding of consent data

For the semantic querying of consent data, the encoding must be in a standardized format. Those queries (e.g., to verify a certain consent status for a selected person-identifier) contain, in addition to person- and project-specific pseudonyms, a description of the person’s consent state. In the first expansion state, this can be based classically on distinct, agreed upon policy identifiers (cf [17]).

The SCC could be an independent option for formulating and processing consent queries (cf [17]), although it allows the description of consent documents regardless of structure and form and supports the practical application of patients’ will to research data. Similarly, this project-independent coding approach was successfully evaluated with respect to the convertibility of existing consent codings into/from the SCC.

### Minimum set of functionalities

Table 4 lists the API functionalities necessary to support the implementation of UC1 (cf. Figure 2) and UC2 (cf. Figure 3). An implementation based on FHIR CRUD Actions and FHIR Search Application Programming Interface (API) is possible. With respect to design decisions, client- and server-based efforts should be determined.

**Table 4:** Overview of required operations for a fTTP consent

Operation (Use Case)	Purpose	IN Parameter	OUT Parameter	Possible Errors
Verify-consent-state (UC1)	consent status inquiry of a particular intended use for several independent pseudonyms (internally under consideration of all aliases)	• project-context • list of overarching pseudonyms • intended action • time of request	• analysis outcome (person-, project-context, reliability of action) as FHIR consent-resources • detailed error messages per cross-site pseudonym • duration of permissions	• consent data unavailable • unauthorized access
Request-consent-state (UC1)	general consent data inquiry for a number of independent person pseudonyms (internally under consideration of all aliases)	• project-context • list of overarching pseudonyms • time of request	• analysis outcome (person-, project-context, reliability of action) as FHIR consent-resources • duration of permissions • detailed error messages per cross-site pseudonym	• consent data unavailable • unauthorized access
Provide-consent-data (UC1)	information about new or updated consent data	• consent data (site-specific pseudonym, project-context, valid/invalid actions, validity period, reference consent template)	• outcome (created, updated, error)	• unknown content (project, template, site-specific pseudonym) • invalid content (site pseudonym, semantic of action, consistency) • unauthorized access
Provide-withdrawal-objection-data (UC2)	information about withdrawals and objections	• site-specific pseudonym • project-context • optional: invalidated actions, validity period, reference template, site identifier	• request confirmation containing the process identifier	• unknown content (project, template, site-pseudonym) • invalid contents (site pseudonym, semantics of action, consistency) • unauthorized access
Check-withdrawal-objection-state (UC2)	inquiry about withdrawals and objections’ progress status	• site-specific pseudonym • project-context • process identifier	• detailed feedback about the process status	• unknown content (project, template, site-pseudonym) • invalid content (site pseudonym, semantics of action, consistency) • unauthorized access
Notify- action-requested (UC2)	notification about a successful action from the ’fTTP Consent’ to a participant (component, site) e.g. received withdrawal or objection	• action type • reason for action • depending on the subject: cross-site pseudonym • process identifier • action identifier • participant identifier (subject) • time stamp	• request confirmation containing the process identifier and task identifier	
Notify-action-resolved (UC2)	notification about an action´s successful completion by the participant (component) to the ’fTTP Consent’	• action identifier • participant identifier (responsible component) • depending on the addresser: cross-site pseudonym, site-specific pseudonym • time stamp • outcome of action • optional: confirmation of action, which person´s withdrawal has been processed within the TTP	• request confirmation containing the process identifier and task identifier	
Notify withdrawal-objection-processed (UC2)	notification of a completed withdrawal/objection process from the ’fTTP Consent’ to the initially reporting site	• site-specific pseudonym • project-context • process identifier • time stamp • process status • list of components and participants who completed the withdrawal or objection	• request confirmation containing the process identifier	

### Facilitating an implementation in federated infrastructures

**Fig. 4:**
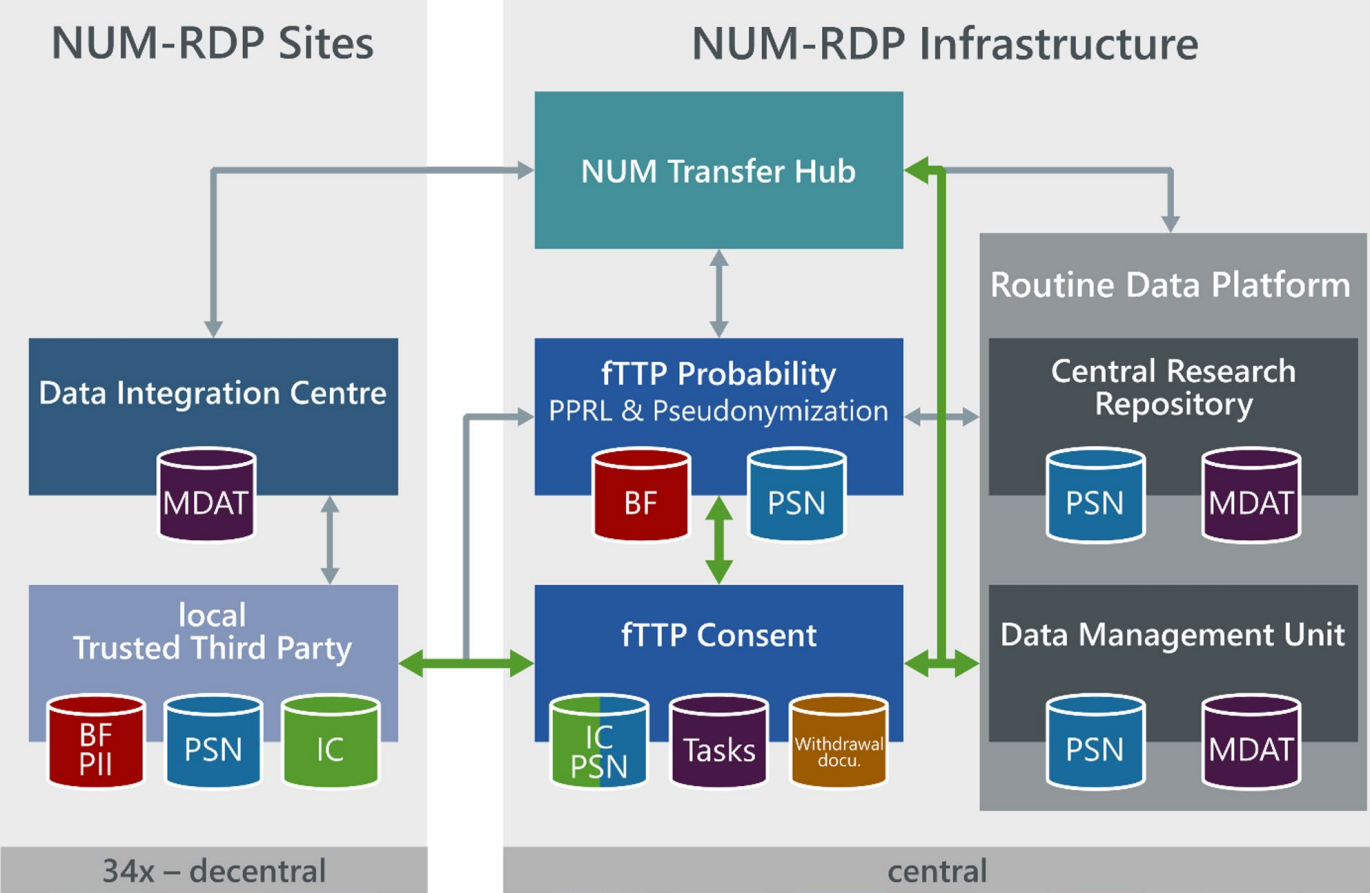
The concept design of ‘fTTP Consent’ aims to facilitate decentralized, federated infrastructures. It focusses on simplifying federated consent and withdrawal/objection processes in the context of cross-site data sharing for research purposes. List of abbreviations used: medical data (MDAT), bloom filter (BF), person-identifying information (PII), pseudonym (PSN), informed consent (IC), privacy-preserving record linkage (PPRL)

As illustrated in Fig. 4 the concept design of the ‘fTTP Consent’ aims to facilitate an implementation in decentralized federated infrastructure. For example, in the NUM RDP with 34 sites (each with local DIC and local TTP) data is managed at the respective site. The record linkage and cross-site pseudonymization of the locally pseudonymized medical data is performed using services of the centrally provided ‘fTTP Probability’ as part of the data transfer process to the Routine Data Platform (please see background section for details). Aligned with that approach, it is intended to link decentral data from consents, withdrawals and objections from the sites. Relevant decentralized consent data are linked centrally in pseudonymized form, to improve data tranfer processes (UC1) and to facilitate central coordination of withdrawal/objection processes (UC2). The concept design of the ‘fTTP Consent’ considered required interfaces to local TTPs, the ‘fTTP Probability’, the NUM Transfer Hub and the Routine Data Platform. As of May 2026, the technical implementation of the ‘fTTP Consent’ concept is planned but pending.

## Discussion

A review of existing preliminary work may give the impression that the concept of consent has generally been considered within the limits of a specific local context or in the context of a particular research endeavor: Technical solutions that facilitate consent management from both perspectives are available [9, 25–27]. Technical formats have been developed to represent structured consent data and its semantic meaning [3, 17, 20, 22, 23, 25]. Modern process standards for the exchange of consent data have been designed [24].

As demonstrated by the MII and the NUM, facilitating federated scenarios in collaborative medical research (with centralized governance) is essential for the sharing of health research data [11]. However, to date, support for federated research scenarios has been limited to privacy-preserving record linkage and pseudonymization [11]. Adding a consent-specific component appears to be the next evolutionary step in transforming existing Trusted Third Party approaches [26, 28] from project-driven or local research to federated research scenarios. To our knowledge, a technical architecture, that combines privacy-preserving record linkage with consent governance focusing on workflow optimization in federated research, has not yet been published.

As elaborated, the ‘fTTP Consent’ concept is able to address the identified needs for improvement by supporting the technical implementation of federated consent, withdrawal and objection processes. Expanding existing fTTP approaches allows for the utilization of established infrastructures and communication links [11]. Thus, only little effort is needed to integrate the participating sites or components (NTH, CRR, DMU).

There are several general requirements for the implementation of an ‘fTTP Consent’ approach, such as an implementation of a technical foundation based on the HL7 FHIR. The same standard is used by the NUM and MII [1]. Opt-in and opt-out approaches (consent, withdrawal, objection) must be supported to allow for a potential expansion to integrate GDNG [6], ePA [7] or the German “Health care Strengthening Act” (Gesundheitsversorgungsstärkungsgesetz, GVSG) [29]. Additionally, the recent developments of WG consent management and opt-in/opt-out profiling work must be considered [20]. Future scenarios and the semantic comparability of consent data must be supported independently from the MII and NUM, e.g., based on an (combination of) SCC [17], ODRL [22] and DPV [23].

The fTTP facilitates efficient communication of the patient’s consent status to its connected institutions and components. This poses not only its greatest advantage but also its greatest challenge. Upon implementation, two requirements must be met. First, all connected components themselves must agree to the use of an ‘fTTP Consent’. If only one party disagrees, the approach will not be implemented successfully. Subsequently, the patient’s rights, e.g., in cases of withdrawal, objection, etc., could be endangered. Second, each participating site must use the same semantic standards for the description of consent data. This poses a great challenge, as individual sites may use varying semantic standards and terminologies. To bridge this gap, the SCC could provide an adequate solution for more standardization in consent semantics across different sites [17].

If all the aforementioned prerequisites are met, the success of a ‘fTTP Consent’ approach still relies on the sites to inform them about an initial consent status change. For decentralized data storage, the responsibility to receive and convey a changed consent status remains with the individual site. It is their mandate to receive potential withdrawals and forward them to ‘fTTP Consent’. This might be a single point of failure, as strongly delayed or error-prone withdrawal and objection notification from the site to the ‘fTTP Consent’ might result in unlawful scientific data usage.

## Conclusions

In this article, complex research scenarios within the context of both opt-in and opt-out legal frameworks were examined. Using the NUM-RDP project as an example, the requirements for correctly taking the patients’ will into account were considered. The proposed solution, ‘fTTP Consent’, was developed to simplify the sharing of consent, withdrawal and objection data, which is essential for legally permissible cross-site data sharing and the linkage of external data sources. The design of the ‘fTTP Consent’ supports both consent-based (opt-in) and legally mandated (opt-out) research scenarios, with a natural focus on the data subject (the patient) and the correct implementation of data subject rights. As the NUM-RDP ended officially in 2025, other research projects can benefit from its upcoming implementation.

The ‘fTTP Consent’ concept is centered on supporting automatable workflows within research infrastructures. The emphasis is on ensuring the correctness and availability of consent data in formal, legal, semantic and syntactic terms. In the future, ‘fTTP Consent’ could be used as an independent, federated consent registry [17] or as a basis for establishing a patient portal. This would facilitate the strengthening of patient-centered, cross-project research. This applies even to pan-European research initiatives such as the European Health Data Space (EHDS) [4].

## Data Availability

Not applicable.

## Abbreviations

API: Application Programming Interface
BF: Bloom Filter
BPMN: Business Process Model and Notation
CODEX: COVID Data Exchange
CRR: Central Research Repository
CRUD: Create, read, update, delete
DIC: Data Integration Centre
DigiG: Act to Accelerate the Digitalization of the Healthcare System (Digital Act)
DMU: Data Management Unit
eCRF: Electronic case report forms
EHDS: European Health Data Space
ePA: Electronic Patient Records
FHIR: Fast Health Care Interoperability Resources
fTTP: federated Trusted Third Party
GDNG: Health Data Utilization Act (Gesundheitsdatennutzungsgesetz)
GDPR: General Data Protection Regulation
GVSG: German “Healthcare Strengthening Act” (Gesundheitsversorgungsstärkungsgesetz)
HL7: Health Level 7
IC: Informed Consent
IHE: Integrating the Health Care Enterprise
MDAT: Medical data
MII: Medical Informatics Initiative Germany
MPI: Master Patient Index
NTH: NUM Transfer Hub
NUM: Network of University Medicine
OIDs: Object Identifiers
PPRL: Privacy-Preserving Record Linkage
PSN: Pseudonym
RDP: Routine Data Platform
SCC: Semantic Consent Code
TFCI: Task Force Consent Implementation
TMF: Technology, Methods and Infrastructure for Networked Medical Research
TTP: Trusted Third Party
WG: Working Group
